# Enhancing the Durability of Bituminous Concrete Using Plastic Waste on Soft Rock Aggregates

**DOI:** 10.3390/polym18070813

**Published:** 2026-03-27

**Authors:** H. Laldintluanga, Zorinkima, Rebecca Ramhmachhuani

**Affiliations:** 1 Department of Civil Engineering, Faculty of Engineering, Mizoram University, Aizawl 796004, India; mzut211@mzu.edu.in; 2 Department of Civil Engineering, Mizoram University, Aizawl 796004, India

**Keywords:** bituminous concrete, LDPE plastic waste, soft rock aggregates, moisture resistance, pavement durability, sustainable road materials

## Abstract

The use of marginal sedimentary aggregates in pavement construction remains a major challenge in mountainous regions due to their high porosity, weak lamination planes, and susceptibility to moisture-induced deterioration. This study investigates the potential of low-density polyethylene (LDPE) plastic waste to enhance the engineering performance of laminated Miocene soft rock aggregates used in bituminous concrete. Aggregates sourced from the Surma Group (Bhuban Formation) in Mizoram, India, were characterized through physico-mechanical, geochemical, and mineralogical analyses to evaluate their durability and moisture sensitivity. X-ray fluorescence (XRF) analysis revealed elevated feldspar and total alkali contents (≈5.15%), indicating a mineralogical composition prone to hydrophilic behavior and stripping within bituminous mixtures. To mitigate these limitations, aggregates were coated with varying proportions of LDPE plastic using the dry process. An optimum LDPE content of 9% by weight of aggregate produced significant improvements in aggregate performance, resulting in a 70.03% reduction in Aggregate Impact Value (from 17.72% to 5.31%), a decrease in Los Angeles Abrasion Value from 42.93% to 31.45%, and an 89.82% reduction in water absorption (from 4.52% to 0.46%). The polymer coating effectively sealed lamination planes and reduced moisture ingress within the sedimentary structure. Bituminous concrete mixtures incorporating LDPE were further evaluated using Marshall stability and indirect tensile strength tests. The addition of 1.1% LDPE by weight of mix significantly enhanced moisture resistance. For mixtures with nominal maximum aggregate sizes (NMASs) of 13 mm and 19 mm, the Tensile Strength Ratio (TSR) increased from 52.59% and 58.58% in the control mixtures to 82.81% and 87.10%, respectively, thereby satisfying the minimum requirement of 80% specified by MoRTH. The results indicate that LDPE functions as a hydrophobic barrier and structural sealant that improves binder–aggregate adhesion and prevents stripping along weak lamination planes. The findings demonstrate that LDPE-modified bituminous concrete provides a sustainable and technically viable strategy for upgrading marginal sedimentary aggregates into durable pavement materials while simultaneously promoting the beneficial reuse of plastic waste.

## 1. Introduction

The rapid expansion of road infrastructure in mountainous terrains, particularly in the Indo-Myanmar range of Northeast India, necessitates the use of locally available construction materials to ensure economic and logistical viability. However, the region is predominantly characterized by the Surma Group (Bhuban Formation), which consists of Miocene sedimentary successions like siltstones, shale, and sandstones. These materials are traditionally classified as marginal aggregates due to their inherent lamination planes, high porosity, and significant moisture susceptibility, which often lead to premature pavement failures such as stripping and raveling. Laminated sedimentary rocks often contain thin bedding planes enriched with clay and silt minerals that act as preferential pathways for water infiltration. When exposed to repeated traffic loading under moist conditions, these weak lamination planes can promote aggregate disintegration and stripping of the bituminous binder from the aggregate surface. Moisture-induced damage in asphalt mixtures is one of the major causes of premature pavement deterioration worldwide, leading to forms of distress such as raveling, cracking, and pothole formation [1]. This problem becomes more severe in high-rainfall regions such as Mizoram, where annual precipitation frequently exceeds 2500 mm, and pavements are subjected to prolonged moisture exposure. To utilize such soft rock effectively, its inherent weaknesses in strength, hardness, and resistance to weathering must be mitigated through innovative material modification.

In recent years, increasing attention has been directed toward the use of waste plastic materials in asphalt mixtures as a sustainable engineering solution. The accumulation of non-biodegradable plastic waste has become a major environmental concern globally, prompting researchers to explore alternative recycling strategies within the construction industry. Among various polymeric wastes, low-density polyethylene (LDPE) is widely available and has favorable thermoplastic properties that make it suitable for asphalt modification. Several studies have reported that the incorporation of waste plastics in asphalt mixtures can significantly improve mechanical properties such as stability, rutting resistance, and moisture durability while simultaneously reducing environmental pollution associated with plastic disposal [2,3,4]. To address this degradation, the incorporation of plastic waste offers a dual benefit technical approach. Beyond the environmental advantages of managing non-biodegradable waste, the application of molten plastic to coat soft-rock aggregates serves as a protective barrier. This coating improves the aggregate impact resistance and specific gravity while significantly reducing water absorption—directly counteracting the moisture-induced crumbling [5,6,7,8,9,10].

However, the effectiveness of this plastic-modified mix is heavily dependent on the skeleton of the aggregate itself. Material gradation and Nominal Maximum Aggregate Size (NMAS) directly influence how the coated particles pack together and distribute stress [11]. While finer gradations can enhance mastic cohesion, coarser gradations in soft rocks often exhibit superior resistance to moisture damage and aggregate degradation due to their reduced surface area and increased individual particle strength [12,13,14]. Therefore, the present study investigates the potential of utilizing low-density polyethylene (LDPE) plastic waste to enhance the durability of bituminous concrete prepared using locally available Miocene soft rock aggregates. It further addresses a critical engineering gap: the structural and chemical inadequacy of Miocene sedimentary rocks for pavements.

Although numerous studies have investigated plastic-modified asphalt mixtures, most of the existing research has focused on hard igneous aggregates such as granite or basalt. Limited attention has been given to laminated sedimentary aggregates, which possess distinct geological characteristics such as high feldspar content, clay-rich matrices, and well-developed lamination planes. These features significantly influence the moisture sensitivity and durability of asphalt mixtures prepared using such materials. Geological studies on the Surma Group sandstones of Mizoram indicate that these rocks contain abundant feldspar and clay minerals derived from weathering processes, which contribute to their relatively high water absorption and reduced mechanical strength [15,16,17].

The research integrates geochemical characterization of the aggregates with laboratory evaluation of aggregate performance and asphalt mixture properties. By examining the effects of LDPE coating on aggregate durability and moisture resistance, the study aims to establish a sustainable engineering approach for upgrading marginal sedimentary aggregates into durable pavement materials suitable for high-rainfall mountainous environments.

## 2. Materials and Methods

### 2.1. Materials

#### 2.1.1. Aggregates

The coarse and fine aggregates used in this study were obtained from laminated sedimentary sandstone belonging to the Surma Group (Bhuban Formation) in Mizoram, India. These rocks represent typical Miocene sedimentary deposits widely used as construction materials in the region due to the limited availability of high-quality aggregates.

Aggregate samples were collected from four quarry locations around Aizawl—Hlimen, Melthum, Sakawrtuichhun, and Zemabawk—to ensure representative sampling of the locally available materials. The collected rocks were crushed and processed into coarse and fine aggregates in accordance with the gradation requirements for bituminous concrete mixtures. Stone dust obtained from the same parent rock was used as the mineral filler in the asphalt mixtures.

#### 2.1.2. Bituminous Binder

Viscosity Grade 30 (VG-30) bitumen was used as the binder material in the preparation of bituminous concrete mixtures. The binder was supplied by Indian Oil Corporation Limited (IOCL), Guwahati, Assam, India. The physical properties of the bitumen, including penetration, softening point, ductility, and specific gravity, were determined in accordance with the specifications of IS 73:2013 [18] to ensure compliance with standard requirements for road construction.

#### 2.1.3. Plastic Modifier

Low-density polyethylene (LDPE) waste was used as the polymer modifier in this study. The plastic waste was collected from a municipal solid waste collection facility in Aizawl, Mizoram. The collected plastic materials were cleaned, shredded into small pieces, and sieved to obtain a uniform particle size suitable for mixing with heated aggregates. LDPE was selected due to its thermoplastic properties, hydrophobic behavior, and compatibility with bituminous materials.

### 2.2. Methods

#### 2.2.1. Geochemical and Mineralogical Analysis

To investigate the chemical characteristics of the aggregates, representative sandstone samples from four quarry sites were prepared in accordance with ASTM D5121 [19] and analyzed using X-ray Fluorescence (XRF). This analysis determined the major oxide compositions, specifically SiO_2_, Al_2_O_3_, Fe_2_O_3_, CaO, MgO, Na_2_O, and K_2_O.

The geochemical evaluation of the sandstone aggregates was performed using two standardized indices: the Chemical Index of Alteration (CIA) and the Index of Compositional Variability (ICV). The CIA, was employed to quantify the degree of chemical weathering. Complementarily, the mineralogical maturity was evaluated using the Index of Compositional Variability (ICV) approach [20,21]. These indices provide a repeatable, mathematically grounded framework for characterizing the susceptibility of soft rock aggregates to moisture-induced degradation, supplementing traditional physical tests.

#### 2.2.2. Physical Property Testing of Aggregates

The engineering properties of the aggregates were evaluated using standard laboratory tests. Both uncoated and LDPE-coated aggregates were tested to assess the influence of plastic modification on aggregate performance. All tests were conducted in accordance with the procedures specified in IS 2386 (Part III and IV) [22,23].

The following tests were conducted:

(i) The Aggregate Impact Value (AIV) test to evaluate the toughness of the aggregates;
(ii) The Los Angeles Abrasion Value (LAAV) test to determine resistance to abrasion and wear;
(iii) Specific gravity and water absorption tests to evaluate aggregate density and porosity;
(iv) An aggregate soundness test using sodium sulfate (Na_2_SO_4_) to assess resistance to weathering.

#### 2.2.3. Gradation

**Figure 1:**
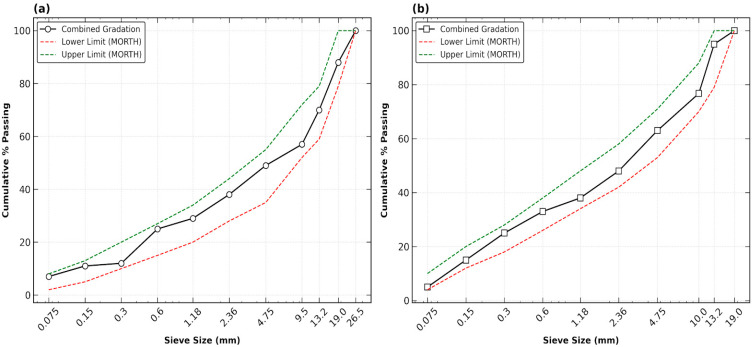
Gradation Curves for (**a**) BC Grade-I and (**b**) BC Grade-II.

Two distinct gradations were adopted based on MoRTH (Table 500-18) standards [24] to investigate the influence of the aggregate skeleton on performance. As illustrated in Figure 1, both gradations fall within the specified limits but provide different surface area to volume ratios. The 13 mm gradation represents a denser, finer matrix, while the 19 mm gradation provides a coarser stone-to-stone contact skeleton. The two gradings used in this study are:

(i) Grading-1 (19 mm NMAS), representing a coarser stone-to-stone contact skeleton;
(ii) Grading-2 (13 mm NMAS), representing a denser, finer matrix.

#### 2.2.4. Preparation of Modified Bituminous Concrete

Bituminous concrete specimens were prepared using the dry mixing process, which involves coating the heated aggregates with plastic prior to the addition of bitumen. The optimum plastic content for aggregate coating and asphalt mixture modification was determined through experimental evaluation. The preparation procedure consisted of the following steps:

(i) Aggregates were heated to a temperature of 150–170 °C to remove moisture.
(ii) Shredded LDPE plastic was added to the hot aggregates and mixed thoroughly to ensure uniform coating of the aggregate particles.
(iii) VG-30 bitumen heated to 150–165 °C was added to the plastic-coated aggregates and mixed until complete coating of the mixture was achieved.
(iv) The mixture was compacted into Marshall specimens at a temperature of 120–145 °C using the standard Marshall compaction method in accordance with ASTM D6927 [25].

#### 2.2.5. Performance and Durability Testing

The performance of the prepared bituminous concrete mixtures was evaluated through mechanical and moisture susceptibility tests.

(i) Marshall Stability and Flow: To evaluate the load-bearing capacity and deformation characteristics;
(ii) Water Sensitivity Analysis: Moisture susceptibility was quantified using the Indirect Tensile Strength (ITS) test (ASTM D6391) [26] and the Tensile Strength Ratio (TSR) to determine the retained strength after moisture conditioning.

## 3. Results and Discussions

### 3.1. Geochemical Analysis of Rock

The durability of bituminous concrete is fundamentally governed by the resistance of its constituent aggregates to weathering and physical disintegration. The results for the four sandstone aggregate sources—Hlimen, Melthum, Sakawrtuichhun, and Zemabawk reveal a consistent failure to meet the standard durability requirements as shown in Table 1. As per IS: 2386, the maximum permissible weight loss after five cycles is limited to 12%. The failure of these aggregates in the soundness test suggests that they are prone to stripping and crushing under traffic loads. In the absence of modification, these materials would compromise the lifespan of a pavement. The observed level of material instability highlights the critical need for this investigation to ensure long-term structural performance.

The geochemical characterization of the rock samples, derived from XRF analysis, is presented in Table 2 and Table 3. This includes summary statistics and key indices such as the Chemical Index of Alteration (CIA) to assess weathering intensity and the Index of Compositional Variability (ICV) to evaluate mineralogical maturity.

The Chemical Index of Alteration (CIA) values ranging from 50 to 65 indicate moderate chemical weathering, reflecting the environmental conditions prevailing during rock formation and suggesting a mineralogical composition rich in feldspars, micas, and lithic fragments. The Index of Compositional Variability (ICV), which is used to assess sediment maturity, exceeds 1.0 for all samples, signifying geochemically immature sediments. Such high ICV values are characteristic of rocks containing substantial proportions of plagioclase, K-feldspar, and ferromagnesian minerals. The fragility and crumbling nature of the aggregate are dominated by its physical lamination and the inherent weakness of the natural cementing material, rather than geochemical weathering, which remains medium as indicated by the CIA value.

The relatively low SiO_2_/Al_2_O_3_ ratios (3.7–4.5), compared with those of stable cratonic sandstone (>10), further confirm the textural and chemical immaturity of the material. The rocks are enriched in iron and magnesium (approximately 9–11%) and exhibit a high alumina content (15–17%), indicating a significant abundance of lithic fragments, a mineralogical signature typical of greywacke-type sediments.

The lower SiO_2_ content (<65%) implies reduced mechanical toughness and increased moisture sensitivity, while the relatively high Al_2_O_3_ content (~16%) and ICV values (>1) point to abundant feldspar and clay minerals that can promote clay swelling under wet conditions. In addition, moderate total alkali content (Na_2_O + K_2_O ≈ 5.15%), dominated by potassium derived from K-feldspar, suggests moderate moisture susceptibility of the aggregates. The inference of Illite and Kaolinite is strongly supported by the correlation between XRF data and established regional stratigraphy. Geological literature on the Surma Group (Mizoram) characterizes these siltstones and sandstones as being rich in fine-grained matrix clays, specifically Illite and Kaolinite, which are the primary alteration products of the high feldspar content (KAlSi_3_O_8_) identified in our study [27]. The geochemical results indicate that the local rock has a chemical affinity for water, making a plastic barrier a scientific necessity.

### 3.2. Performance Enhancement of Marginal Soft Rock Through LDPE Waste Coating

**Figure 2:**
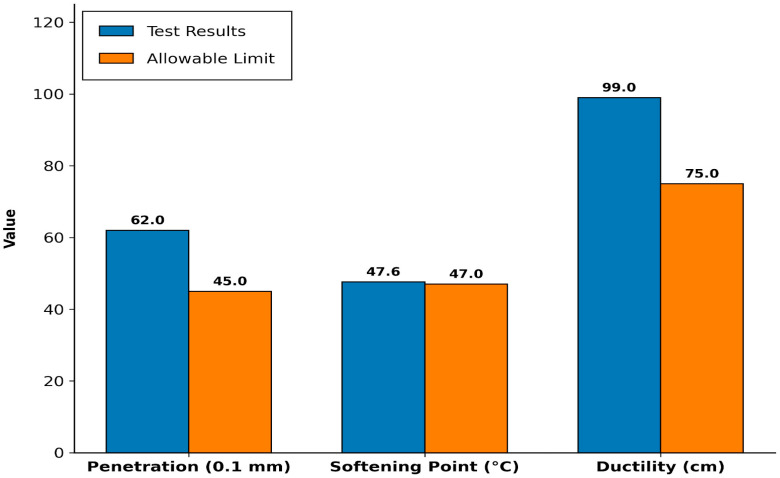
Physical Properties of Bitumen.

The physical properties of the VG-30 bitumen were evaluated to establish a baseline prior to modification, and the results confirmed that the binder satisfies the requirements specified in IS 73:2013, as shown in Figure 2. Although the binder exhibits adequate ductility and adhesion characteristics, its performance in asphalt mixtures containing laminated sedimentary aggregates can be compromised due to the contrasting surface properties of the two materials. Bitumen is inherently hydrophobic, whereas the sandstone aggregates used in this study exhibit hydrophilic behavior due to their relatively high water absorption (4.52%) and the presence of feldspar and clay-rich lamination planes. This mismatch in moisture affinity can weaken the binder–aggregate bond and promote moisture-induced stripping.

**Figure 3:**
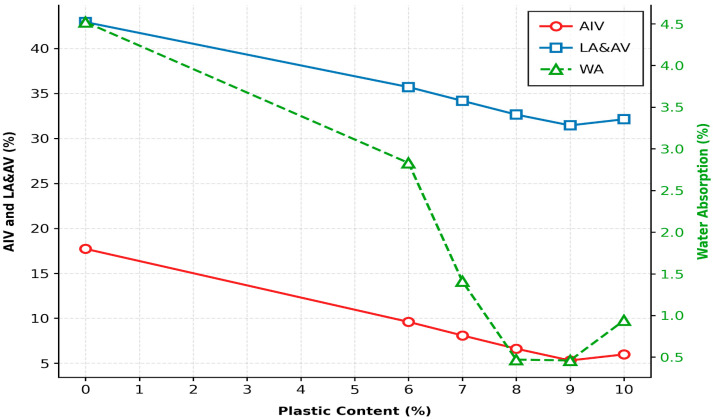
Physical properties of aggregate with and without LDPE plastic coating.

Thinly laminated sedimentary rocks are inherently susceptible to weathering due to the presence of weak lamination planes composed primarily of clay and silt. These layers act as preferential pathways for water ingress, leading to deterioration of interparticle bonding and reduced aggregate strength. As shown in Figure 3, coating the aggregate surface with LDPE plastic significantly reduced impact value, abrasion value, and water absorption.

The plastic coating functions as a binding agent that firmly bonds surface particles of the coarse aggregate, thereby enhancing aggregate toughness (impact resistance), hardness, abrasion resistance, and resistance to water absorption, while simultaneously sealing the lamination planes. The formation of a thin, impermeable plastic film around the aggregate particles restricts moisture penetration, resulting in a progressive reduction in water absorption with increasing plastic content.

The influence of LDPE plastic coating on aggregate physical properties was evaluated across a range of 0% to 10%. The Aggregate Impact Value (AIV) decreased from 17.72% (uncoated) to a minimum of 5.31% at 9% plastic content. Further addition to 10% resulted in a slight increase to 5.98%. The Los Angeles Abrasion Value (LAAV) followed a similar pattern, reaching its maximum resistance (31.45%) at 9% plastic content before showing a slight decline at 10% (32.13%). Water Absorption (WA) saw a drastic reduction from 4.52% in the control sample to 0.46% at 9% plastic addition. This indicates that 9% provides the most efficient coating for sealing the lamination planes of the soft rock. Consequently, the optimum plastic content was identified as 9% by weight of aggregate, as it represents the peak performance threshold across all critical physical parameters.

The significant enhancement in aggregate properties observed in this study aligns with several previous investigations into polymer-coated marginal materials. The reduction in AIV (from 17.72% to 5.31%), LAAV (from 42.93% to 31.45%), and Water Absorption (from 4.52% to 0.46%) achieved at a 9% LDPE content reflects the findings of authors who reported that coating overburnt brick aggregates with waste plastic significantly upgraded their mechanical toughness and reduced permeability [28]. Furthermore, the 9% optimum content identified for laminated soft rock is slightly higher than the 5–7% range typically reported for hard aggregates like granite or basalt [29]. This discrepancy is attributed to the high initial porosity and well-developed lamination planes of the Mizoram sandstone, which require a thicker polymer film to achieve complete encapsulation. Similar observations were made by other researchers, where higher plastic concentrations were necessary to effectively seal the pore networks of relatively porous aggregates [30]. The incorporation of LDPE plastic serves as an intermediate hydrophobic layer that coats the aggregate surface, seals lamination planes, and enhances the adhesive interaction between the binder and the aggregate particles.

### 3.3. Performance of Bitumen Blended with Plastic in Bituminous Concrete

The potential of plastic waste to enhance the performance of weak aggregates used in road construction was evaluated by blending plastic with bitumen. In addition, the effectiveness of plastic-coated aggregates and the influence of plastic–bitumen blending on the properties of bituminous concrete were investigated.

The Optimum Bitumen Content (OBC) was established by synthesizing the peak performance parameters of the Marshall mix design. Specifically, the OBC was calculated as the numerical average of the bitumen percentages yielding maximum bulk density, peak Marshall stability, and the median values for both flow and air voids (Va). Following this multi-criteria optimization, the OBC for Bituminous Concrete (BC) Grade I and Grade II was determined to be 5.3% and 5.5%, respectively.

A major limitation of using plastic as the sole binder is the difficulty in achieving adequate compaction at lower temperatures, as plastic solidifies rapidly upon cooling. Although a Marshall stability value of 12.99 kN was obtained at 11% plastic content, the mixture failed to satisfy standard workability and compaction requirements, rendering plastic unsuitable as the only binding material. Furthermore, it exhibited high values of voids filled with bitumen (VFB) and voids in mineral aggregate (VMA), exceeding the permissible limits specified by MoRTH, as shown in Table 4.

The mechanical and volumetric response of BC-II mixtures to incremental LDPE addition, as presented in Table 5, reveals a clear parabolic trend in Marshall stability, identifying a critical threshold for structural enhancement. The data indicates a progressive increase in Marshall stability as the plastic content rises from 0.28% to 1.10%. The stability value nearly triples from the initial modified state (1682.75 kg) to a peak of 4675.19 kg at 1.1% LDPE addition. This sharp ascent suggests that the LDPE acts as a reinforcing matrix, significantly stiffening the binder and improving the load-sharing capacity between the soft rock aggregate particles. However, exceeding this concentration (at 1.38% plastic) results in a decline to 4400.55 kg, indicating that excessive polymer may interfere with the aggregate-to-aggregate contact, leading to a lubricating effect that destabilizes the mix. These results validate the utilization of plastic waste as a performance-enhancing agent for improving the structural characteristics of bituminous concrete.

### 3.4. Durability (Moisture Sensitivity) Analysis of Bituminous Concrete

Flexible pavements increasingly experience premature deterioration due to moisture-induced damage, which arises from a loss of cohesion within the bituminous mixture caused by weakened adhesion between the binder and aggregate. This phenomenon, commonly referred to as stripping, is a major contributor to pavement distress. The tensile strength ratio (TSR), also known as retained tensile strength, is widely used to evaluate the moisture susceptibility and water resistance of bituminous mixtures. Higher TSR values indicate lower strength loss under moisture conditioning and, consequently, greater resistance to water damage.

Durability (moisture sensitivity) tests were conducted on Bituminous Concrete (BC) Grade II mixtures with nominal maximum aggregate sizes (NMASs) of 19 mm and 13 mm. The aggregates used were found to be inherently susceptible to moisture damage due to their high water absorption, weathering-related degradability, and the presence of lamination planes within the rock structure. Consequently, plastic was introduced to enhance the aggregate and BC mixture performance. According to MoRTH specifications, the minimum required retained tensile strength is 80%, a criterion that was not satisfied by the unmodified mixtures.

**Figure 4:**
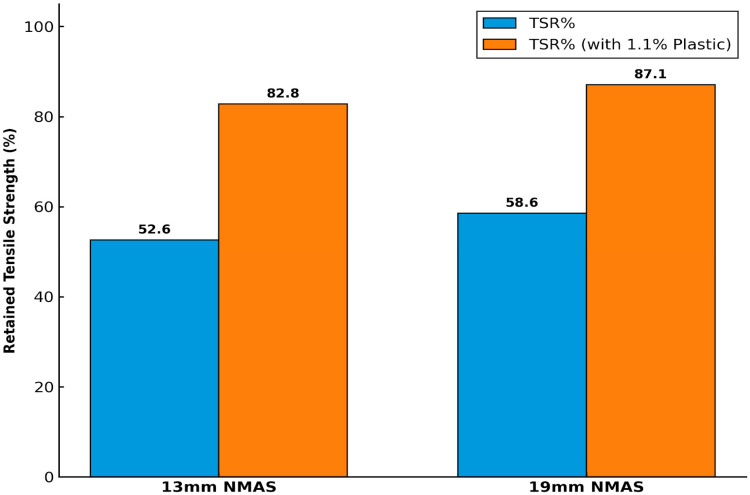
Retained Tensile Strength (%) of BC Mixtures with Different Gradations.

Both gradations exhibited a substantial reduction in tensile strength after exposure to moisture conditioning. As shown in Figure 4, the control mixtures exhibited high moisture susceptibility with TSR values of 52.59% (13 mm NMAS) and 58.58% (19 mm NMAS). These values fall significantly below the MoRTH specification of 80%. This poor performance is geochemically linked to the high alumina and total alkali content (≈5.15%) of the soft rock, which promotes hydrophilic behavior and stripping at the binder aggregate interface. Upon the addition of 1.1% LDPE plastic, a substantial increase in TSR was observed, exceeding the 80% threshold for both gradations. This confirms that the plastic film successfully seals the weak lamination planes identified in the XRF analysis.

To improve moisture resistance, a total binder content of 6.6%, comprising 5.5% bitumen and 1.1% LDPE plastic, was incorporated into the BC mixtures. In the absence of plastic, the retained tensile strength values were 52.59% for the 13 mm NMAS mix and 58.58% for the 19 mm NMAS mix. With plastic, these values increased markedly to 82.81% and 87.10%, respectively, as illustrated in Figure 4, thereby satisfying MoRTH requirements and demonstrating the effectiveness of plastic in enhancing moisture resistance.

The moisture resistance results achieved in this study represent a significant improvement over conventional bituminous mixes and compare favorably with other polymer-modified asphalt studies. For instance, while the inclusion of waste plastic in bituminous mixes has been shown to improve moisture resistance, TSR values typically range between 82% and 88% for standard aggregates [30]. The superior performance observed in this study (TSR of 82.81% to 87.10%) suggests that the addition of LDPE plastic provides a more effective hydrophobic barrier for marginal aggregates than simple binder modification.

This confirms that for marginal, soft aggregates, the physical encapsulation provided by the addition of 1.1% LDPE in the bitumen is the primary mechanism preventing the displacement of bitumen by water. This is particularly critical for the laminated sedimentary rocks, where internal pore water pressure otherwise leads to rapid delamination along the bedding planes. The improvement in aggregate and mixture performance observed in this study can be attributed to the combined mechanical and physicochemical effects of LDPE coating.

### 3.5. Effect of Aggregate Gradation on Mixture Durability

**Figure 5:**
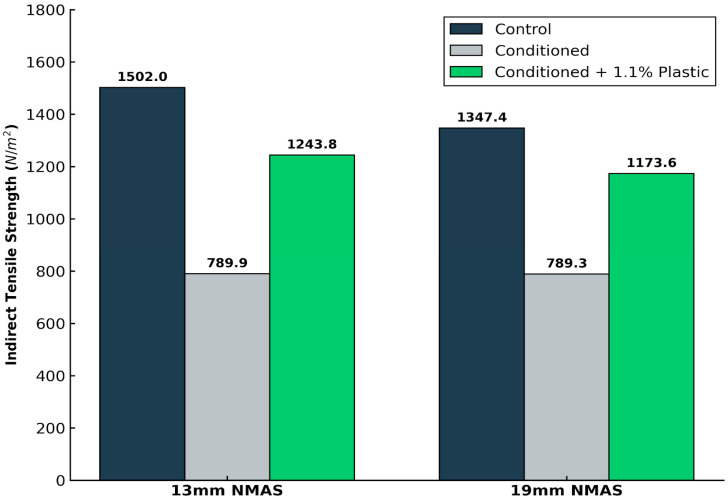
Tensile Strength of BC Mixtures with Different Gradations.

The mechanical response of the bituminous concrete (BC) mixtures under moisture conditioning is intrinsically linked to the internal packing and skeletal structure of the aggregate. A clear performance divergence exists between the 13 mm and 19 mm Nominal Maximum Aggregate Size (NMAS) gradations as illustrated in Figure 5.

The 13 mm NMAS mixtures initially achieved higher tensile strength. This is attributed to the increased surface area of finer particles, which promotes denser packing and enhances internal mastic cohesion. Despite higher initial strength, the 13 mm gradation suffered a greater decline in performance when subjected to moisture conditioning. The finer matrix, while cohesive, possesses a higher total surface area that increases the exposure of hydrophilic mineral surfaces, specifically the alumina-rich clays and feldspars, to water ingress.

In contrast, the 19 mm NMAS gradation provided a more resilient structural skeleton. The coarser stone-to-stone contact reduces the overall surface area vulnerable to stripping and provides a more robust matrix that is less prone to the mechanical degradation common in porous, sedimentary rocks.

## 4. Conclusions

This study investigated the potential of utilizing low-density polyethylene (LDPE) waste to enhance the engineering performance of marginal Miocene soft rock aggregates in bituminous concrete. Based on the experimental results and comparative analysis, the following conclusions are drawn:

The coating of soft rock aggregates with LDPE proved highly effective in mitigating the inherent weaknesses of the sedimentary rock. Waste plastic functions as an effective binding and sealing agent, firmly bonding aggregate particles and sealing lamination planes. This results in enhanced aggregate toughness (impact resistance), hardness, abrasion resistance, and a reduction in water absorption.

A 9% optimum plastic content (by weight of aggregate) was identified as the threshold required to achieve complete surface encapsulation and structural sealing of lamination planes. The application of LDPE modification resulted in a 70.03% reduction in the Aggregate Impact Value (AIV), lowering it from a marginal 17.72% to a superior 5.31%. Furthermore, the Aggregate Crushing Value and Los Angeles Abrasion resistance improved by 31.29% and 38.83%, respectively, transforming the soft rock into a high-performance pavement material. Plastic contents exceeding 9% by weight of aggregate do not provide additional performance benefits. The use of plastic as the sole binder is not recommended due to inadequate workability and failure to meet performance requirements.

The addition of 1.1% LDPE (by weight of mix) in a bituminous concrete significantly enhanced moisture resistance. The Tensile Strength Ratio (TSR) showed a relative increase of 52.59% for the 13 mm NMAS gradation and 58.58% for the 19 mm NMAS gradation, with final values reaching 82.81% and 87.10%, respectively, both exceeding the 80% MoRTH regulatory threshold.

Bituminous concrete with a finer aggregate gradation (13 mm NMAS) exhibits higher tensile strength due to denser particle packing, whereas mixtures with a coarser gradation (19 mm NMAS) demonstrate higher retained tensile strength, primarily owing to reduced aggregate degradation within the bituminous matrix.

The required level of retained tensile strength in bituminous concrete was successfully achieved by incorporating 1.1% plastic with 5.5% bitumen (by weight of mix). The combined use of plastic and larger aggregate sizes substantially improves moisture resistance and wheel-load performance, making laminated sedimentary aggregates suitable for pavement applications. The study confirms that LDPE-modified bituminous concrete offers a scientifically viable and sustainable pathway for recycling plastic waste while simultaneously addressing the scarcity of high-quality road aggregates.

## Figures and Tables

**Table 1:** Rock Durability Assessment Using Soundness Testing.

Source of Rock	Percentage (%) Loss of Weight After 5 Cycles	Permissible Limit as per IS 2386
Hlimen	15.55	<12% with Sodium sulphate
Melthum	16.27	
Sakawrtuichhun	17.17	
Zemabawk	14.23	

**Table 2:** XRF-Based Rock Geochemical Analysis.

Source of Rock	Na_2_O	MgO	Al_2_O_3_	SiO_2_	P_2_O_5_	SO_3_	K_2_O	CaO	TiO_2_	MnO	Fe_2_O_3_
Hlimen	2.56	4.32	15.74	65.62	0.16	0.61	2.58	1.4	0.95	0.05	5.75
Melthum	2.25	4.57	16.84	63.53	0.12	0.67	2.88	1.75	0.94	0.06	6.16
Sakawrtuichhun	2.48	4.47	16	62.41	0.18	1.01	2.67	3.07	1.08	0.08	6.29
Zemabawk	2.79	4.19	14.89	67.8	0.16	0.27	2.42	1.28	0.95	0.04	5

**Table 3:** Key Geochemical Indices.

Source of Rock	CIA	ICV	SiO_2_/Al_2_O_3_	Al_2_O_3_/TiO_2_	K_2_O/Na_2_O	Fe_2_O_3_ + MgO
Hlimen	63.2	1.12	4.17	16.57	1.01	10.07
Melthum	63.42	1.11	3.77	17.91	1.28	10.73
Sakawrtuichhun	59.15	1.26	3.9	14.81	1.08	10.76
Zemabawk	61.93	1.12	4.55	15.67	0.87	9.19

**Table 4:** Marshall Characteristics of BC-II with 100% LDPE Plastic Waste as Binder.

BC-II (Only Plastic)	% Plastic	Vv	Vb	VMA	VFB	Stability in kg	Flow mm
S1	7	8.1	15.2	23.3	65.3	697	4
S2	9	1.6	20.4	22	92.9	866.9	3
S3	11	1.4	24.4	25.9	94.5	1299.9	3.2
S4	13	1.3	28.3	29.6	95.7	1192	4.1
S5	15	1.2	32	33.2	96.4	942.7	5
S6	17	1	35.6	36.7	97.2	845.2	5.3
S7	20	0.9	40.8	41.7	97.8	433.4	6

**Table 5:** Marshall Properties of BC-II with Incremental LDPE Plastic Waste.

BC Grade II	% Binder	Vv	Vb	VMA	VFB	Stability in kg	Flow mm
S1 (5.5% + 0.28% Plastic)	5.78	5.6	13.11	18.71	70.07	1682.75	3.5
S1 (5.5% + 0.55% Plastic)	6.05	7.87	13.35	21.22	62.9	3803.33	3
S1 (5.5% + 0.83% Plastic)	6.33	8.27	13.85	22.12	62.62	4314.83	2.5
S1 (5.5% + 1.1% Plastic)	6.6	7.88	14.45	22.34	64.71	4675.19	3.2
S1 (5.5% + 1.38% Plastic)	6.88	6.97	15.16	22.13	68.51	4400.55	3.3

## Data Availability

All data generated or analyzed during this study are included in this published article.

## Abbreviations

The following abbreviations are used in this manuscript:

AIV	Aggregate impact value
ASTM	American Society for Testing and Materials
BC	Bituminous concrete
CIA	Chemical Index of Alteration
ICV	Index of Compositional Variability
IS	Indian Standard
ITS	Indirect tensile strength
LAAV	Los Angeles abrasion value
LDPE	Low-density polyethylene
MoRTH	Ministry of Road Transport and Highways
NMAS	Nominal maximum aggregate size
SG	Specific gravity
Vb	Volume of bitumen
VFB	Void filled with bitumen
VMA	Void in Mineral aggregate
Vv	Volume of void
WA	Water absorption
XRF	X-ray fluorescence
